# Balancing Efficiency and Accuracy in Hepatitis C Rapid Antibody Testing: Insights From a Cluster Randomised Crossover Trial

**DOI:** 10.1111/jvh.70043

**Published:** 2025-07-03

**Authors:** K. Heath, R. Guzman, I. Elsum, A. J. Wade, K. Allardice, J. Kasza, M. Bryant, A. J. Thompson, M. Stoové, T. Snelling, N. Scott, T. Spelman, D. A. Anderson, J. Richmond, J. Howell, N. Andric, P. Dietze, P. Higgs, R. Sacks‐Davis, A. Forbes, M. E. Hellard, A. E. Pedrana, J. S. Doyle

**Affiliations:** ^1^ Burnet Institute Melbourne Victoria Australia; ^2^ Monash University School of Public Health and Preventive Medicine Melbourne Victoria Australia; ^3^ St. Vincent's Hospital Melbourne Victoria Australia; ^4^ University of Sydney Sydney New South Wales Australia; ^5^ The Alfred Department of Infectious Diseases Monash University Melbourne Victoria Australia

**Keywords:** diagnostic accuracy, hepatitis C, people who inject drugs, point‐of‐care, rapid antibody test

## Abstract

Hepatitis C remains a significant global health problem, particularly among people who inject drugs. To achieve Australia's 2030 elimination targets, efficient testing strategies are needed. The OraQuick rapid antibody test provides results in 20 min, but many non‐viraemic individuals with resolved infections test positive, potentially leading to unnecessary confirmatory RNA testing. Reducing the read time to five minutes has been proposed to reduce false positives and improve efficiency, but its impact on viremia detection is unclear. This study utilised data from the QuickStart study, a randomised controlled trial investigating different rapid testing pathways and a same‐day test‐and‐treat model. Participants underwent OraQuick rapid antibody testing with results read at both five and 20 min, followed by confirmatory RNA testing. Among 298 participants with OraQuick and RNA test results, the 20‐min OraQuick test was positive for all 79 viraemic individuals and 156 non‐viraemic individuals. At five minutes, positive results decreased to 77 (97.5%) of viraemic and 135 (87%) non‐viraemic individuals with positive 20‐min results. Using a five‐minute result to trigger RNA testing would have reduced unnecessary RNA testing by 13% in our cohort at the cost of missing 2.5% of viraemic individuals. A five‐minute read time could improve efficiency by reducing unnecessary RNA testing, but confirmatory RNA testing remains essential to distinguish active from resolved infections. The balance between efficiency and accuracy may vary contextually, reflecting differing rates of resolved infections. This approach may be beneficial in resource‐limited settings, but the potential for missed viremia must be considered.

**Trial Registration:** ClinicalTrials.gov number; NCT05016609

## 1 Introduction

Hepatitis C is a major public health problem; an estimated 50 million people live with chronic viral infection globally [1]. In 2016, the World Health Organization (WHO) set elimination targets for hepatitis C, including treatment of 80% of chronic cases [2, 3]. However, only 11 countries, including Australia, are on track to reach elimination targets by 2030 [4].

Since 2016, Australia has funded unrestricted access to direct‐acting antivirals (DAAs), irrespective of disease stage or risk behaviour, prompting rapid treatment uptake. However, testing and treatment rates have since declined, necessitating new strategies to engage the remaining individuals with hepatitis C [5, 6].

People who inject drugs are at elevated risk of hepatitis C infection, comprising an estimated 8.5% of infections and with an estimated 830,000 new infections annually attributed to injecting drug use [7, 8]. Globally, an estimated 47% (nationally, 0%–93%) of people who report injecting drugs in the past 12 months have been tested for hepatitis C antibodies [9]; in Australia, 40% of this group are estimated to have viraemic infection [7]. Most Australians who inject drugs are tested for hepatitis C antibodies in their lifetime, but around 32% of people with hepatitis C who inject drugs have never received treatment [9, 10, 11].

Diagnosis of hepatitis C is typically multi‐step; venous blood samples are screened for antibodies before reactive samples undergo ribonucleic acid (RNA) testing [12, 13]. The delay between venepuncture and laboratory results can result in loss to follow‐up, compounded in people who inject drugs by additional barriers such as venous access challenges [14]. In one Australian cohort, venous sampling was difficult in 48% of participants with a history of injecting drug use [15]. Alternative sampling methods, including rapid tests, offer a promising solution.

Australia has taken steps to simplify hepatitis C testing, including approval of a rapid RNA test (2021) and a rapid antibody test (2024) by the Australian Register of Therapeutic Goods (ARTG) [16]. These tests can be performed outside a laboratory, with minimal (Cepheid GeneXpert RNA) or no equipment (bioLytical INSTI antibody), and without extensive operator training [16]. A national rollout programme (2022 to 2026) has subsidised 50,000 GeneXpert RNA tests [17]. By March 2022, over 22,000 tests were conducted, including 16,452 RNA tests with a 15.6% positivity rate, leading to 1645 treatments [18].

While rapid GeneXpert RNA testing is highly accurate, it requires up to 60 min for a result [13, 19]. In contrast, rapid antibody testing can be completed within minutes and may serve as a useful screening tool before RNA confirmation, particularly in resource‐limited settings [20].

The OraQuick rapid antibody test, a single‐use lateral‐flow indirect immunoassay with a 20‐min read time, was pre‐qualified for professional use by the WHO in 2017 and self‐test in 2024, but is not yet ARTG‐approved. In pooled and real‐world cohorts in Canada and Madrid, reducing the OraQuick read time from 20 min to five reduced the detection of non‐viraemic individuals, while maintaining 100% sensitivity for viraemic patients [21]. This suggests that a five‐minute read time could improve efficiency by reducing unnecessary RNA tests for non‐viraemic individuals. Further studies have highlighted the utility of rapid antibody testing to infer hepatitis C viremia in treatment‐naïve populations [19]. However, the feasibility of this approach for hepatitis C elimination efforts in cohorts with resolved infections or prior treatment remains uncertain due to the potential for false positives.

The QuickStart study is a randomised controlled trial of three rapid testing pathways and a novel same‐day test‐and‐treat model on time to treatment among people who inject or have injected drugs [22]. This study, nested within the QuickStart study, aims to assess the utility of a five‐minute OraQuick rapid antibody test to infer hepatitis C viremia from high antibody titres. We considered two hypotheses: (1) OraQuick test results read at five minutes would concord with those read at 20 min, and (2) OraQuick test results read at five minutes would concord with hepatitis C RNA test results. In evaluating the trade‐off between efficiency and diagnostic accuracy, this analysis seeks to inform future hepatitis C testing strategies, particularly in settings where streamlined diagnosis could enhance case detection and treatment uptake.

## 2 Methods

### 2.1 Study Design

The QuickStart study is a clustered randomised crossover trial with three intervention arms and one standard of care arm [22]; recruitment finished at the end of 2024, with follow‐up due to finish in May 2025. In brief, the QuickStart study's primary objective is to assess the effects of the rapid testing pathways and a novel same‐day test‐and‐treat model on hepatitis C treatment uptake and cure. Arm A uses OraQuick rapid antibody testing with standard of care. Arm B adds reflexive GeneXpert RNA testing for antibody‐positive results. Arm C initiates same‐day treatment with DAAs for antibody‐positive results. The control arm follows standard of care without rapid testing, including confirmatory laboratory testing [12]. OraQuick test was administered because it is widely used, easy to administer, and suitable for point‐of‐care settings.

### 2.2 Sub‐Study: Five‐Minute OraQuick Rapid Antibody Testing

The OraQuick rapid hepatitis C antibody test for fingerstick capillary blood samples provides results in around 20 min. Arms A, B and C use rapid, point‐of‐care OraQuick antibody testing with one reading at five minutes and another at the recommended 20 min. The GeneXpert rapid RNA, used by Arm B only, detects hepatitis C RNA using fingerstick capillary blood to provide results in around 60 min.

All participants are invited to undergo venepuncture for confirmatory laboratory testing, with the point‐of‐care result presented as preliminary. The number of participants with RNA test results (laboratory or GeneXpert) was anticipated to be lower than the number with point‐of‐care antibody results because participants can decline confirmatory laboratory testing; people who inject or have injected drugs can have poor venous access that prevents confirmatory testing; only Arm B uses GeneXpert rapid RNA testing; and because the study continues, some laboratory RNA results are yet to be recorded.

The sub‐study was restricted to data from participants with complete rapid antibody testing and matched RNA test results (laboratory or GeneXpert) by 10 September 2024. The sub‐study did not disaggregate data by arm because it was concerned only with the concordance between test results.

### 2.3 Site and Participant Eligibility

The QuickStart study is being conducted at Australian metropolitan and rural primary healthcare services with a general practitioner and/or a nurse practitioner experienced in testing and treating hepatitis C and high caseloads of people who currently or previously injected drugs and are at risk of hepatitis C infection.

The study includes participants who have injected drugs at least once, are aged 18+ years, are attending a participating primary healthcare clinic, have not had DAA treatment, are Medicare eligible and can speak and understand English. The study excludes individuals known to be currently pregnant or breastfeeding, currently engaged in hepatitis C treatment, unable to provide informed consent, tested for hepatitis C in the past three months, and/or who had previous successful hepatitis C treatment with interferon‐based therapy.

### 2.4 Data Collection and Processing

Data were collected using a REDCap electronic data capture tool hosted at the Burnet Institute [23, 24]. Survey questions used to collect the data analysed in this sub‐study are shown in Tables S1 and S2. Demographic data were collapsed into condensed categories, as shown in Table S1.

RNA test results from GeneXpert and laboratory tests were combined into a single variable, with laboratory results prioritised in cases of disagreement or GeneXpert error, and the available result used when only one test was conducted.

### 2.5 Data Analysis

Our analyses used two sub‐cohorts of data from the QuickStart study. Sub‐cohort 1 included participants with five‐ and 20‐min OraQuick rapid antibody results. Sub‐cohort 2, a subset of sub‐cohort 1, included participants who also had RNA test results. Demographics in each sub‐cohort were summarised using counts and frequencies, and χ^2^ testing compared the demographics of sub‐cohorts. Participant records were excluded from χ^2^ testing if they lacked demographic data or if their demographic category had fewer than five participants.

We conducted three initial analyses, comparing the performance of the:

1. Five‐minute and 20‐min OraQuick rapid antibody tests in sub‐cohort 1.
2. Five‐minute OraQuick rapid antibody test relative to RNA testing (GeneXpert or laboratory) in sub‐cohort 2.
3. Twenty‐min OraQuick rapid antibody test relative to RNA testing (GeneXpert or laboratory) in sub‐cohort 2.

Using the true positive $\left(\mathrm{TP}\right)$, true negative $\left(\mathrm{TN}\right)$, false positive $\left(\mathrm{FP}\right)$ and false negative $\left(\mathrm{FN}\right),$ we calculated test sensitivity $\left(\mathrm{TP}/\left(\mathrm{TP}+\mathrm{FN}\right)\right)$, specificity $\left(\mathrm{TN}/\left(\mathrm{TN}+\mathrm{FP}\right)\right)$, positive predictive value (PPV) $\left(\mathrm{TP}/(\mathrm{TP}+\mathrm{FP}\right))$ and negative predictive value (NPV) $\left(\mathrm{TN}/\left(\mathrm{TN}+\mathrm{FN}\right)\right)$. Invalid results and testing errors were excluded from calculations of predictive accuracy. Confidence intervals were calculated using the normal approximation, as outlined in the Supporting Information.

A fourth analysis compared the need for confirmatory RNA testing with the five‐minute and 20‐min OraQuick rapid antibody test read times. The fold‐difference in the number of reflexive RNA tests required was estimated by comparing the proportion of valid OraQuick tests with positive results at each read time. The fold‐difference in unnecessary RNA testing was estimated by comparing the proportion of positive OraQuick tests with a negative confirmatory RNA result at each read time.

## 3 Results

### 3.1 Description of the Study Cohort

Sub‐cohort 1 had 647 participants, and sub‐cohort 2 had 298. Demographics of study sub‐cohorts are shown in Table 1. Around two‐thirds of each sub‐cohort had injected drugs in the six months before enrolment. Around one‐third of each sub‐cohort was experiencing houselessness or in other informal living arrangements. In both sub‐cohorts, 98% reported no previous hepatitis C treatment. Previous hepatitis C diagnosis was the only variable significantly different between sub‐cohorts, with 34% of sub‐cohort 1 having had a positive hepatitis C antibody and/or RNA test, versus 59% in sub‐cohort 2.

**TABLE 1:** Demographic data, presented by sub‐cohorts used in analysis. Sub‐cohort 1 included participants with five‐ and 20‐min OraQuick rapid antibody results. Sub‐cohort 2, a subset of sub‐cohort 1, included participants who also had RNA test results. Some participants were later deemed ineligible to continue within the QuickStart study (e.g., due to prior treatment with DAAs); for this sub‐study, these participants were included if they had data on hepatitis C testing.

	Sub‐cohort 1	Sub‐cohort 2	*χ* ^2^ test result
Accommodation and housing currently lived in			
Private residential housing	161 (25%)	78 (26%)	*p* = 0.25
Public housing or supported accommodation	261 (40%)	133 (45%)	
Informal or less stable housing	157 (24%)	64 (21%)	
Experiencing houselessness	59 (9%)	18 (6%)	
Other or no data	9 (1%)	5 (2%)	
Age			
Under 30 years old	46 (7%)	13 (4%)	*p* = 0.05
30–44 years old	293 (45%)	121 (41%)	
45 years and older	307 (47%)	164 (55%)	
No data	1 (< 1%)	0 (< 1%)	
Self‐reported injecting drug use in the past six months			
Yes	425 (66%)	203 (68%)	*p* = 0.56
No	206 (32%)	89 (30%)	
No data	16 (2%)	6 (2%)	
Highest level of education attained			
Primary school or less	43 (7%)	22 (7%)	*p* = 0.30
High school	413 (64%)	187 (63%)	
TAFE/technical qualification	124 (19%)	67 (22%)	
Diploma, undergraduate or postgraduate	64 (10%)	20 (7%)	
No data	3 (< 1%)	2 (1%)	
Current employment status			
Full‐time	71 (11%)	29 (10%)	*p* = 0.76
Part‐time	63 (10%)	27 (9%)	
Not employed, incl. studying or home duties	483 (75%)	230 (77%)	
No data or other	30 (5%)	12 (4%)	
Gender identity			
Female	234 (36%)	113 (38%)	*p* = 0.64
Male	408 (63%)	182 (61%)	
Other gender identity	3 (< 1%)	2 (1%)	
No data	2 (< 1%)	1 (< 1%)	
Previous HIV diagnosis			
Yes	5 (1%)	1 (< 1%)	*p* = 0.73
No	637 (98%)	294 (99%)	
No data	5 (1%)	3 (1%)	
Previous positive hepatitis C diagnosis			
Yes—positive antibody and/or RNA	221 (34%)	177 (59%)	*p* < 0.01
No	405 (63%)	112 (38%)	
No data	21 (3%)	9 (3%)	
Previous hepatitis C treatment			
Yes—direct‐acting antivirals	2 (< 1%)	0 (< 1%)	
Yes—pegylated interferon	4 (1%)	3 (1%)	
No	636 (98%)	292 (98%)	
No data	5 (1%)	3 (1%)	
Previous history of incarceration, being on remand or in police cells			
Yes	376 (58%)	186 (62%)	*p* = 0.24
No	259 (40%)	107 (36%)	
No data	12 (2%)	5 (2%)	
Sex assigned at birth			
Female	235 (36%)	115 (39%)	*p* = 0.56
Male	411 (64%)	183 (61%)	
No data or other	1 (0%)	0 (0%)	

### 3.2 Analysis 1: Performance of OraQuick Rapid Antibody Test Results at Five Minutes Relative to 20 Min in Sub‐Cohort 1

Five of the 647 participants in sub‐cohort 1 had invalid or unreadable OraQuick rapid antibody results at five and/or 20 min (Table 2), so they were excluded from analysis. Of the remaining 642 participants, 283 had positive OraQuick results at 20 min; a five‐minute read identified 253 of these with 89% sensitivity. Of the 283 participants with a positive result at 20 min, 11% (*n* = 30) had a negative result at five minutes. All 359 participants with negative OraQuick results at 20 min were identified by a five‐minute read with 100% specificity.

**TABLE 2:** Comparison of OraQuick rapid hepatitis C antibody test results read at five minutes with the same test read at 20 min. Invalid and unreadable results were excluded from calculations of predictive accuracy.

	OraQuick rapid antibody test result at 20 min		
	Positive	Negative	Invalid
OraQuick rapid antibody test result at five minutes			
Positive	253	0	0
Negative	30	359	0
Invalid	1	3	1
Test failure rate (OraQuick at 5 min)	0.9%		
Test failure rate (OraQuick at 20 min)	0.4%		
Sensitivity (95% CI)	89.4% (85.8%–93.0%)		
Specificity (95% CI)	100% (100%–100%)		
Positive predictive value (95% CI)	100% (100%–100%)		
Negative predictive value (95% CI)	92.3% (89.6%–94.9%)		

### 3.3 Analysis 2: Performance of OraQuick Rapid Antibody Test Results at Five Minutes Relative to RNA Test Results in Sub‐Cohort 2

Of the 298 participants in sub‐cohort 2, 95 (32%) had GeneXpert rapid RNA test results, of which two (2%) conflicted with laboratory RNA test results and six (6%) produced errors; 271 (91%) had laboratory results in lieu of or in addition to GeneXpert results. Table S4 shows participants in sub‐cohort 2 according to their results from GeneXpert and laboratory testing.

Four of the 298 participants in sub‐cohort 2 had unreadable OraQuick rapid antibody results at five minutes or RNA testing errors (Table 3), so they were excluded from analysis. Of the remaining 294 participants, 79 had positive RNA results. Of these, 77 also had positive five‐minute OraQuick results, meaning the rapid antibody test indicated viremia with 98% sensitivity. Of the 215 participants with negative RNA results, 80 had negative OraQuick results at five minutes, meaning the rapid antibody indicated the absence of viremia with 37% specificity.

**TABLE 3:** Comparison of OraQuick rapid hepatitis C antibody test results read at five minutes with hepatitis C RNA test results using GeneXpert or laboratory testing (see Methods). Invalid results, unreadable results and test errors were excluded from calculations of predictive accuracy.

	RNA test result (either GeneXpert or laboratory testing)		
	Detected	Not detected	Error
OraQuick rapid antibody test result at five minutes			
Positive	77	135	1
Negative	2	80	0
Invalid	0	3	0
Test failure rate (OraQuick at 5 min)	1.0%		
Test failure rate (GeneXpert & lab tests)	0.3%		
Sensitivity (95% CI)	97.5% (94.0%–100%)		
Specificity (95% CI)	37.2% (30.8%–43.7%)		
Positive predictive value (95% CI)	36.3% (29.9%–42.8%)		
Negative predictive value (95% CI)	97.6% (94.2%–100%)		

### 3.4 Analysis 3: Performance of OraQuick Rapid Antibody Test Results at 20 Min Relative to RNA Test Results in Sub‐Cohort 2

Two of the 298 participants in sub‐cohort 2 had unreadable OraQuick rapid antibody results at 20 min or RNA testing errors (Table 4), so were excluded from analysis. Of the remaining 296 participants, all 79 with positive RNA results also had positive OraQuick results, meaning the rapid antibody test indicated viremia with 100% sensitivity. Of the 217 participants with negative RNA results, 61 had negative OraQuick results, meaning the rapid antibody indicated the absence of viremia with 28% specificity.

**TABLE 4:** Comparison of OraQuick rapid hepatitis C antibody test results read at 20 min with hepatitis C RNA test results using GeneXpert or laboratory testing (see Methods). Invalid results, unreadable results and test errors were excluded from calculations of predictive accuracy.

	RNA test result (either GeneXpert or laboratory testing)		
	Detected	Not detected	Error
OraQuick rapid antibody test result at 20 min			
Positive	79	156	1
Negative	0	61	0
Invalid	0	1	0
Test failure rate (OraQuick at 5 min)	0.3%		
Test failure rate (GeneXpert & lab tests)	0.3%		
Sensitivity (95% CI)	100% (100%–100%)		
Specificity (95% CI)	28.1% (22.1%–34.1%)		
Positive predictive value (95% CI)	33.6% (27.6%–39.7%)		
Negative predictive value (95% CI)	100% (100%–100%)		

### 3.5 Analysis 4: Confirmatory RNA Testing Requirements

The five‐minute OraQuick rapid antibody read time yielded fewer positive results than the 20‐min read time (Table 5), reducing the number of confirmatory RNA tests needed by approximately 9%. Additionally, the five‐minute test yielded fewer positive results that were not indicative of hepatitis C RNA, reducing the number of unnecessary confirmatory RNA tests by approximately 13%.

**TABLE 5:** Confirmatory RNA test requirements of five‐minute versus 20‐min OraQuick rapid antibody testing in sub‐cohort 2, in which participants had both rapid antibody and RNA results.

	Positive rapid antibody tests that would require confirmatory RNA testing	Confirmatory RNA tests that would yield negative results
Five‐minute OraQuick test; see Table 3	212 (72.1% of 294 valid tests administered)	135 (45.9% of 294 valid tests administered)
20‐min OraQuick test; see Table 4	235 (79.4% of 296 valid tests administered)	156 (52.7% of 296 valid tests administered)
Fold‐difference between five and 20‐min read times	0.91	0.87

## 4 Discussion

Our results highlight a clinical trade‐off between reducing unnecessary reflexive RNA testing and ensuring accurate viremia detection. Reducing the OraQuick rapid antibody test read time from 20 min to five would have reduced unnecessary reflexive RNA testing by approximately 13% in our cohort, at the cost of failing to detect around 2.5% of viraemic participants. With the five‐minute OraQuick test, 64% of participants with detected antibodies were non‐viraemic, showing the need for confirmatory RNA testing to differentiate active from resolved infections.

Declining hepatitis C testing rates necessitate re‐evaluation of the testing and treatment paradigm. Rapid antibody tests, such as the OraQuick test, are adaptable, with a five‐minute read time proving a useful diagnostic complement to the 20‐min test. A positive OraQuick result at five minutes triggers reflexive RNA testing, while individuals without detectable hepatitis C antibodies at five minutes but with them at 20 min are likely non‐viraemic and do not require further RNA testing [21]. This dual approach could streamline diagnosis, increase throughput in large screening programmes, and lower costs through reducing unnecessary RNA testing for non‐viraemic individuals. Our data indicate that, in our cohort, this approach would identify and administer RNA testing to 97.5% of viraemic individuals and 87% of non‐viraemic individuals with residual antibodies, with the remaining individuals not receiving RNA testing.

The difference between five‐ and 20‐min OraQuick test readings reflects antibody levels in blood samples. Hepatitis C antibodies, produced in response to viral replication, are higher in active infection than in past or waning infections, and higher antibody titres correlate with hepatitis C viremia [25]. Antibody titres decline in the absence of viral antigen following cure, but do not diminish immediately [26]. In rapid testing, antibodies are typically detected earliest in individuals with current viremia or recent cure. In clinical and real‐world cohorts in Canada and Madrid, all 227 viraemic patients had positive OraQuick results within five minutes [21]. Lower circulating antibody titres in individuals with long‐term resolved infections may require the OraQuick test's recommended 20 min for a positive result.

In our cohort, a five‐minute OraQuick test would eliminate 9% of all RNA testing and 13% of unnecessary RNA testing of non‐viraemic participants with residual hepatitis C antibodies (likely from previously resolved infections). However, rates of previously treated and/or spontaneously cleared infections will vary between study populations. In mixed cohorts, the specificity of rapid antibody testing for indicating viremia has varied, likely driven by variable rates of prior infection [21]. About one‐third of hepatitis C infections are thought to clear spontaneously, which may have been underestimated historically [27].

Our study indicated a notable rate of previously resolved infections, with 66% of positive 20‐min OraQuick tests being confirmed as RNA negative. While spontaneous clearance of hepatitis C is estimated at 28%–36% in Australia, factors like genetics and co‐infections may influence this rate in subpopulations, making it difficult to determine its precise contribution in our cohort [11]. In addition, underreporting of previous treatment may contribute to the rate of previously resolved infections given the evidence of social desirability response bias, fragmented healthcare experiences, and reduced health literacy in our study population [28]. With 10% of treated individuals who have undergone retreatment, the likelihood of prior treatment may be higher in our cohort than in other countries, even if not accurately recalled [29]. These findings highlight the need for context‐sensitive rapid testing, particularly in marginalised groups like people who inject drugs, and underscore the need for further research on the rate of previously resolved infections.

In populations with complex health needs and retention challenges, the utility of a testing approach goes beyond its predictive accuracy. People who inject drugs often face concurrent health issues and negative past healthcare experiences [30]. Five‐minute rapid antibody testing administered by nurses at the point of care provides prompt, visible results and expedites reflexive RNA testing, which may increase engagement. The increased acceptability of non‐venous testing in people who inject drugs may increase participation further [31]. Rapid point‐of‐care tests facilitate screening in accessible locations such as needle and syringe exchange programmes, and enable innovative care models tailored to people who inject drugs. For example, the QuickStart study is exploring a “same‐day test‐and‐treat” approach: two‐week DAA starter packs are dispensed based on positive five‐minute OraQuick results, with further treatment dispensed following confirmatory lab results [22]. Small‐scale trials have shown that this model increases cure rates compared to standard referral methods [32].

Our study acknowledges certain limitations and assumptions. First, although literature points to the retention benefits of shorter testing durations, we lacked the data to test this hypothesis. Second, rapid testing has advanced since the launch of the QuickStart study. Although our sub‐study did not evaluate new rapid antibody tests such as the one‐minute INSTI antibody test, our findings are relevant for guiding integration of such tests into clinical practice. The effectiveness of five‐ and 20‐min OraQuick read times in balancing efficiency and accuracy is underpinned by the test's antibody detection threshold at these read times. Emerging rapid antibody tests will vary in their detection limits and their ability to support additional diagnostic steps, such as multiple read times, which can be evaluated against our findings for the OraQuick test. Third, our data cannot establish whether invalid point‐of‐care test results using OraQuick or GeneXpert arose through user error or the testing platforms themselves.

Our results highlight the potential of a five‐minute read time as an adjunct to the 20‐min OraQuick test, triggering RNA testing for positive results at five minutes and assuming resolved infections for results positive only at 20 min. This approach offers efficiency benefits that must be balanced against the possibility of missing a small proportion of viraemic individuals with a five‐minute OraQuick test. Reflexive RNA testing remains essential alongside rapid antibody testing to differentiate active from resolved infections. Ongoing evaluation and adaptation of testing protocols are important to identify their context‐specific considerations, maximise their utility in priority populations and remain on track to eliminate hepatitis C.

## Author Contributions

All authors made significant contributions to the design and/or conception of the QuickStart study. K.H., J.S.D. and A.E.P. conceived of this paper's research questions. K.H., R.G., K.A., A.J.W., J.S.D., A.E.P. and M.E.H. made significant contributions to the interpretation of the analysis. All authors made contributions to the technical discussion of the results. All authors critically reviewed the work, provided intellectual content and gave final approval for the version to be published. All authors agree to be accountable for the work, ensuring that questions related to the accuracy or integrity of the work are properly investigated.

## Disclosure

Trial Progression: The study commenced recruitment on 9 March 2022 and is expected to complete recruitment in December 2024.

## Ethics Statement

The study has been approved by the Alfred Ethics Committee (number HREC/64731/Alfred‐2020‐217,547). Each participant will provide written informed consent. Reportable adverse events will be reported to the reviewing ethics committee.

## Conflicts of Interest

J.S.D., M.E.H. and A.E.P. report investigator‐initiated funding to their institution from Gilead Sciences, AbbVie and Merck. A.J.W. reports investigator‐initiated funding to her institution from Gilead Sciences. A.E.P. reports honoraria for educational events from Gilead Sciences, and J.S.D. reports honoraria to his institution from Gilead Sciences and AbbVie. P.H. reports investigator‐initiated funding to their institution from Gilead Sciences and AbbVie. J.H. reports honoraria from Roche Diagnostics and Astra‐Zeneca unrelated to this study, and investigator‐initiated funding from Gilead Sciences unrelated to this study.

## Supporting information


Data S1.

## Data Availability

Research data are not shared.
