# Supplementary material for: Balancing Efficiency and Accuracy in Hepatitis C Rapid Antibody Testing: Insights From a Cluster Randomised Crossover Trial
Source: J Viral Hepat. 2025 Jul 3;32(8):e70043. doi: 10.1111/jvh.70043 (PMC12224559; doi:10.1111/jvh.70043)
Supplement: Supplementary file 1 — Data S1. [file JVH-32-0-s001.docx]

**Supplementary Information**

Contents

[Demographic survey questions analysed in the sub-study 1](#_Toc176948420)

[Clinical survey questions analysed in the sub-study 3](#_Toc176948421)

[Calculation of diagnostic accuracy 4](#_Toc176948422)

[Combination of rapid GeneXpert® and laboratory RNA test results 4](#_Toc176948423)

# **Demographic survey questions analysed in the sub-study**

The sub-study described in this paper used a subset of the survey questions employed by the QuickStart study. The survey questions in this subset used to collect demographic data are shown in Supplementary Table 1. Post-processed of collected data was undertaken to improve ease of data interpretation and to increase statistical power for χ^2^ testing to detect differences in the distribution of demographics between sub-cohorts.

Supplementary Table 1. Survey questions used to collect demographic data and details of post-processing undertaken on demographic data.

|  | **Survey question** | **Possible responses** | **Post-processing** |
| --- | --- | --- | --- |
| Q1. Accommodation | What type of accommodation does the participant currently live in? | - Boarding (e.g. paying for accommodation with family/friends) - Boarding house - Couch surfing - Experiencing houselessness - Living with friend or family without paying rent - Owner occupied property - Rental property (private) - Rental property (public) - Supported accommodation - Squat - Other | Responses were reclassified into the following categories:   - *‘Private residential housing’*, which included: - Owner occupied property - Rental property (private) - *‘Public housing or supported accommodation’*, which included: - Rental property (public) - Supported accommodation - *‘Informal or less stable housing’*, which included: - Boarding [..] - Boarding house - Couch surfing - Living with friend or family [..] - Squat - *‘Experiencing houselessness’* - *‘No data or other’*, which included: - Other - Any participant records without data for Q1 |
| Q2. Age | What is the participant’s date of birth? | Calendar date of format DD-MM-YYYY | Age was calculated as the difference between date of birth and the date of data extract (24-06-2024). Ages were then classified into the following categories:   - *‘Under 30 years old’* - *‘30-44 years old’* - *‘45 years and older’* - *‘No data’*, which included any participant records without data for Q2 |
| Q3. Self-reported intravenous drug use | In the past six months, has the participant injected drugs? | - Yes - No - Prefer not to say | The response ‘Prefer not to say’ was reclassified as ‘*No data*’, alongside participant records without data for Q3 |
| Q4. Education | What is the highest grade/year of education the participant has completed? | - Primary school - High school - TAFE/technical qualification or TAFE qualification at certificate level - Advanced diploma or diploma - Bachelor degree - Graduate diploma or graduate certificate - Postgraduate degree - Has not completed formal schooling | Responses were reclassified into the following categories:   - *‘Primary school or less’*, which included: - Primary school - Has not completed formal schooling - *‘High school’* - *‘TAFE/technical qualification’* - *‘Diploma, undergraduate or postgraduate’*, which included: - Graduate diploma or graduate certificate - Advanced diploma or diploma - Bachelor degree - Postgraduate degree - *‘No data’*, which included any participant records without data for Q4 |
| Q5. Employment | What is the participant’s current employment status? | - Not employed - Full-time - Part-time - Student - Home duties - Other | Responses were reclassified into the following categories:   - *‘Full-time’* - *‘Part-time’* - *‘Not employed, incl. studying or home* duties’, which included: - Not employed - Student - Home duties - *‘No data or other’*, which included: - Other - Participant records without data for Q5 |
| Q6. Gender identity | What is the participant’s gender identity? | - Male - Female - Trans Man - Trans Woman - Indigenous Brotherboy - Indigenous Sistergirl - Gender non-conforming - Other - Prefer not to say | Responses were reclassified into the following categories:   - *‘Male’* - *‘Female’* - *‘Other gender identity*’, which included: - Trans Man - Trans Woman - Indigenous Brotherboy - Indigenous Sistergirl - Gender non-conforming - Other - *‘No data’*, which included: - Prefer not to say - Participant records without data for Q6 |
| Q7. HIV | Has the participant ever received a positive HIV diagnosis? | - Yes - No - Don’t know | The response ‘Don’t know’ was reclassified as ‘*No data*’, alongside participant records without data for Q7 |
| Q8. Hepatitis C diagnosis | Has the participant previously had a positive hepatitis C test? | - Yes – positive hepatitis C antibody and/or hepatitis C RNA - No - Don’t know | The response ‘Don’t know’ was reclassified as ‘*No data*’, alongside participant records without data for Q8 |
| Q9. Hepatitis C treatment | Has the participant had any previous treatment with direct acting antivirals or pegylated interferon/ribavirin? | - Yes – direct acting antivirals - Yes – pegylated interferon/ribavirin - No - Don’t know | The response ‘Don’t know’ was reclassified as ‘*No data*’, alongside participant records without data for Q9 |
| Q10. Incarceration | Has the participant ever been incarcerated in prison, in remand, or in police cells? | - Yes - No - Don’t know - Prefer not to say | The responses ‘Don’t know’ and ‘Prefer not to say’ were reclassified as ‘*No data*’, alongside participant records without data for Q10 |
| Q11. Sex assigned at birth | What sex was the participant assigned at birth? | - Male - Female - Intersex - Prefer not to say | The responses ‘Intersex’ and ‘Prefer not to say’ were reclassified as ‘*No data or other*’, alongside participant records without data for Q11 |

# **Clinical survey questions analysed in the sub-study**

The survey questions used to collect data on hepatitis C test results are shown in Supplementary Table 2. OraQuick® rapid antibody testing was offered to participants in Arms A, B and C; GeneXpert® rapid RNA testing was offered in Arm B; and all arms offered laboratory antibody and RNA testing, although laboratory antibody results were not included in this sub-study.

Supplementary Table 2. Survey questions used to collect hepatitis C test results and details of where post-processing was applied to collected data.

|  | **Survey question** | **Possible responses** | **Post-processing** |
| --- | --- | --- | --- |
| Q1. OraQuick® rapid antibody results at five minutes | What was the rapid hepatitis C antibody test result at five minutes? *Asked only of participants in Arms A, B and C* | - Positive - Negative - No visible result | .. |
| Q2. OraQuick® rapid antibody results at 20 minutes | What was the rapid hepatitis C antibody test result at 20 minutes? *Asked only of participants in Arms A, B and C* | - Positive - Negative - No visible result - Invalid | .. |
| Q3. GeneXpert® rapid RNA results | What was the rapid hepatitis C RNA test result?  *Asked only of participants in Arm B* | - Detected - Not detected - Invalid - Error - N/A – test not done | Q3 was combined with Q4 to create a single variable as outlined in the main text. |
| Q4. Laboratory RNA results | What was the laboratory RNA test result?  *Asked of participants in all arms* | - Positive - Negative | Q3 was combined with Q4 to create a single variable as outlined in the main text. |

# **Calculation of diagnostic accuracy and confidence intervals.**

For each comparison in our study, a 2x2 table can be constructed, dichotomising results into test positive or negative as show in Supplementary Table 3.

Supplementary Table 3. Example table comparing test results and reference standard procedure, where TP=true positive, FP=false positive, FN=false negative, and TN=true negative.

|  |  | **Reference standard procedure** | |
| --- | --- | --- | --- |
|  |  | **Positive** | **Negative** |
| **Test result** | **Positive** | TP | FP |
|  | **Negative** | FN | TN |

Sensitivity and 95% confidence intervals on sensitivity, were calculated as follows:

$Sensitivity=\frac{TP}{TP+FN}$ ( 1 )

${SE}_{sensitivity}=\frac{\sqrt{sensitivity(1-sensitivity)}}{TP+FN}$ ( 2 )

$95\% confidence interval=sensitivity\pm1.96(\mathrm{SE}_{\mathrm{sensitivity}})$ ( 3 )

Specificity and 95% confidence intervals on specificity, were calculated as follows:

$Specificity=\frac{TN}{TN+FP}$ ( 4 )

${SE}_{specificity}=\frac{\sqrt{specificity(1-specificity)}}{TN+FP}$ ( 5 )

$95\% confidence interval=specificity\pm1.96({SE}_{specificity})$ ( 6 )

Positive Predictive Value (PPV) and 95% confidence intervals on PPV, were calculated as follows:

$PPV=\frac{TP}{TP+FP}$ ( 7 )

${SE}_{PPV}=\frac{\sqrt{PPV(1-PPV)}}{TP+FP}$ ( 8 )

$95\% confidence interval=PPV\pm1.96({SE}_{PPV})$ ( 9 )

Negative Predictive Value (NPV) and 95% confidence intervals on NPV, were calculated as follows:

$NPV=\frac{TN}{TN+FN}$ ( 10 )

${SE}_{NPV}=\frac{\sqrt{NPV(1-NPV)}}{TN+FN}$ ( 11 )

$95\% confidence interval=NPV\pm1.96({SE}_{NPV})$ ( 12 )

# **Combination of rapid GeneXpert® and laboratory RNA test results**

In sub-cohort 2, GeneXpert® rapid RNA test results and laboratory RNA test results were combined to create one variable denoting the combined RNA test result. The methods for combining results from these two tests are given in the main text. Participants in sub-cohort 2 are stratified by their results from the GeneXpert® and rapid RNA tests in Supplementary Table 4.

Of the 298 participants in sub-cohort 2, 95 (32%) had GeneXpert® rapid RNA test results, with or without confirmatory laboratory testing. Among these, two (2%) conflicted with laboratory RNA results, and six (6%) produced errors. Additionally, 27 (28%) of the GeneXpert® results lacked confirmatory laboratory tests, including one participant with a testing error. The majority of sub-cohort 2 (n=269, 90%) either had agreement between error-free GeneXpert® results and laboratory results, or had only laboratory results available.

Supplementary Table 4. Combination of GeneXpert® rapid RNA test results and laboratory RNA test results.

| **GeneXpert® rapid RNA test result** | **Laboratory RNA test result** | **Number of participants** | **Combined RNA test result** |
| --- | --- | --- | --- |
| Detected | Detected | 20 | Detected |
| Detected | Not detected | 1 | Not detected |
| Detected | No result | 5 | Detected |
| Not detected | Detected | 1 | Detected |
| Not detected | Not detected | 41 | Not detected |
| Not detected | No result | 21 | Not detected |
| Error | Detected | 0 | Detected |
| Error | Not detected | 5 | Not detected |
| Error | No result | 1 | Testing error |
| No result | Detected | 53 | Detected |
| No result | Not detected | 150 | Not detected |
